# Workers’ experiences of healthy work environment indicators at well-functioning primary care units in Sweden: a qualitative study

**DOI:** 10.1080/02813432.2018.1523987

**Published:** 2018-09-27

**Authors:** Kristina Areskoug Josefsson, Gunilla Avby, Monica Andersson Bäck, Sofia Kjellström

**Affiliations:** a School of Health and Welfare, Jönköping Academy for Improvement of Health and Welfare, Jönköping University, Jönköping, Sweden;; b Department of Social Work, University of Gothenburg, Gothenburg, Sweden

**Keywords:** Occupational health, organisational performance, primary care, joy at work

## Abstract

**Objective:** Staff experiences of healthy work environment (HWE) indicators at primary care units can assist in understanding why some primary care units function better than others. The aim of the study was to create increased understanding of how workers experienced HWE indicators at well-functioning primary care units.

**Design:** Fifty in-depth interviews with staff at six primary care units in Sweden were analysed with deductive content analysis, revisiting a systematic review of HWE indicators.

**Results:** The study presents additional perspectives on staff experiences of HWE indicators at well-functioning primary care units. The included primary care units (PCU) shared a similar pattern of work environment indicators, with unique solutions and strategies to meet shared challenges. Staff at the included PCUs were encouraged to work to create and sustain a HWE, but each domain (indicator) also provided challenges that the staff and organisation needed to meet. The results suggest that useful approaches for a healthy work environment could be to address issues of organisational virtuousness, employee commitment and joy at work.

**Conclusions:** Both managers and staff are encouraged to actively work not only to create and sustain an HWE but also to promote organisational virtuousness, employee commitment, joy at work and to increase the performance at work, which is of benefit to staff, patients and society.

**Key Points**
- Staff at well-functioning primary care units (PCUs) experienced healthy work environments
- The included PCUs shared a similar pattern of work environment indicators, with unique solutions and strategies to meet shared challenges.
- Staff at the included PCUs were encouraged to work to create and sustain a healthy work environment, but each domain (indicator) also provided challenges that the staff and organisation needed to meet.
- The results suggest that useful approaches for a healthy work environment could be to address issues of organisational virtuousness, employee commitment and joy at work.

## Introduction

Primary care is under pressure to meet patient demands and societal expectations, which raises the urgent question of how it is possible to have healthy working conditions in daily work. Swedish primary care is no exception, and has undergone several changes since 2010, including implementation of a more flexible system that allows patients to choose between private and publicly managed primary care providers [1,2]. The changes also included the introduction of a new form of performance target intended by decision makers to drive specific provider behaviours, even though research has shown the difficulty of making accurate predictions [3]. Primary care has traditionally been a public monopoly based upon a value ground of equity, with a shift of power since the 1980s resulting in a decentralised healthcare system [4–6]. Despite these changes, there are still several common problems in Swedish primary health care, such as waiting times for consultation, lack of patient centredness, and uneven distribution of services [4]. In addition, research on Swedish primary care has mentioned negative changes after implementing the performance target. Negative changes that have been described include an increased number of visits to primary health care physicians, especially by patients in more affluent groups and with lower health care needs, less provision of service for patients with complex needs and a more strained work situation for nurses and managers [5–8]. Increased work demands, job strain from reduced work control and moral strain stand out as important risk factors for an unhealthy work environment [9].

A healthy work environment (HWE) has often been described focusing on physical, psychosocial and organisational conditions affecting the health of the employees, and often from the perspective of the present problems in an organisation [10]. HWE is grounded in intentional, systematic and collaborative efforts, with a culture, climate and practice that create an environment promoting employee health and safety, as well as nurturing organisational effectiveness regarding profit and production [11,12], a process that tends to require changing long-standing cultures, traditions and hierarchies [13]. There is a broad variety of indicators of HWE, which are often intertwined and combined with individual, organisational and societal perspectives, making the workplace important [10,11]. The HWE indicators explored in this study are: positive, accessible and fair leaders; skilled communication; collaboration/teamwork; positive social climate; employee involvement; autonomy/empowerment; role clarity with clear expectations and goals; recognition; growth and development of the individual at work; moderate work pace and workload; administrative and/or personal support at work; safe physical work; good relations with stakeholders.

Workers’ experiences and a positive perception of the workplace may alter the evaluation of work characteristics, even if the work environment remains unchanged [14,15]. An HWE may prevent negative consequences affecting the individual’s health in the workplace and may have a promotional effect on the mental health and well-being of the workers [11,16], thus indicating the importance of establishing and maintaining an HWE for both the organisation and the individual.

There is relatively little direct empirical research on HWE [17], suggesting that there is a need for increased knowledge of how HWE factors are experienced in primary care. Given that the primary care has many demands to handle, we designed a project to study well-functioning primary health care units to see if and how they experienced healthy working conditions.

### Objectives

The aim of this study was to create increased understanding of how workers experienced HWE indicators at well-functioning primary care units (PCUs).

## Study population and methods

The study has a qualitative design and uses a deductive methodology.

### Sample

A common setting or organisation of a Swedish primary care unit is 4–10 physicians specialising in general practice, working with several other healthcare professionals, such as nurses (and specialist nurses), physiotherapists, occupational therapists, social workers and cognitive therapists [2,4]. The professional role of managers at PCUs varies. Six well-functioning PCUs from one county in Sweden were carefully chosen to be included in this study [18]. The purposive sampling focused on well-functioning PCUs, defined as PCUs providing high-quality care according to national and regional comparisons (e.g. national patient surveys), low staff turnover, positive financial development, and good leadership. The PCUs were chosen by the researchers in collaboration with the county’s unit for primary care results. The PCUs were:

- PCU A: a large, public, well-established urban unit, with a stable patient list and a dependable long-term manager.
- PCU B: a large, urban, private unit established at the time of the reform by the manager and five physicians, and now increased in size.
- PCU C: a large, public, urban unit, with staff reductions implemented due to a serious financial situation when the reform was introduced. It had worked hard to get physicians in place to improve the situation.
- PCU D: a small, family owned, private and rural unit, in operation for 15 years before the reform. The reform allowed the unit to increase in size and implement ideas, visions and innovation. It had one official manager, but in practice, leadership was shared between the official manager and a partnering physician.
- PCU E: a large, public, urban unit in the process of changing negative results and personnel turnover, particularly physicians, into an improved situation.
- PCU F: a small, non-profit, urban unit, started at the time of the reform by a group of physicians. It had a good reputation, but a long period with no leader and leadership difficulties resulted in informal leaders and economic problems.

The sample had equal proportions of female and male managers.

### Data collection

The manager of each PCU suggested participants for the study, aiming for participants varying in occupational role, age, gender, years of work experience and years of work experience at the PCU. Informed consent was given by each participant. In total, 50 semi-structured interviews took place at the PCUs and each lasted approximately one hour (Table 1). The interview guide had a broad scope, covering work environment, leadership, innovation and work motivation. The interviews were audio-recorded and transcribed verbatim.

**Table 1.** Occupational role of the participants at the PCUs.

Occupational role	PCU A	PCU B	PCU C	PCU D	PCU E	PCU F
Administrative staff	2	2	3	1	1	2
Physicians	3	2	2	1	1	2
Nurses	3	4	3	1	2	2
Assistant nurses	1		1		1	
Occupational therapists		1		2	1	
Physiotherapists		1		1	2	1
Other				1		
Total	9	10	9	7	8	7

### Data analysis

The analysis had a deductive design [19] and was performed in four steps using a pre-constructed analysis matrix of HWE indicators (Table 2): (1) analysis of each interview; (2) compilation of the results for each PCU; (3) analysis of the results compiled for each PCU; (4) comparison of the main findings for the PCUs. Each step of the analysis was performed by one of the researchers, and then checked with the researcher who performed the interviews. The research group then had analytical discussions to achieve consensus. All the authors were experienced senior qualitative researchers and none of the researchers had previous work or research relations to any of the included PCUs.

**Table 2.** Deductive matrix of HWE indicators.

Healthy work place indicators	Examples of quotes
Positive, accessible and fair leaders	She is the best boss I have ever had; she is outstanding.
Skilled communication	We have managed to create an environment where we do not actually whine. I do not know when I heard whining lately, so if you pick up something for discussion, yes, you may complain in pairs among ourselves, but not openly.
Collaboration/teamwork	Everybody is helpful and you do this in everyone's best interests, not for your own sake, but for everyone.
Positive social climate	We have recruited those who have, what shall I say, so to speak a gleam in the eyes and a will to do something. So that is what distinguishes us from the previous organizations I have worked in.
Employee involvement	I think it is great fun if we reach the goals, you are looking like wow now we got over 75% on this, now we have got a full pot. That way, it is great fun.
Autonomy/empowerment	There is a shared responsibility in the group and you expect everyone to take their responsibility.
Role clarity with clear expectations and goals	If you have several tasks in your role, like I have had three for a while, it divides your attention so much that it does not work so well.
Recognition	I have always liked it and enjoyed challenges. You may feel that it is hard to do it, but once you have done it, you feel satisfied and feel ‘Oh! I did it’, and then you get huge energy from this.
Growth and development of the individual at work	I have a great opportunity to develop in what I think is interesting.
Moderate work pace and workload	There is not quite enough time to do it in a good way. Yes, that bothered me a lot. I may not have time to reflect fully or check things out as much as I would like. And I also cannot take those extra seconds needed to make the meeting with the patient a little more personal.
Administrative and/or personal support at work	I get full support from the manager, so we discuss how to do it together.
Safe physical work	There is a lack of space and so, really we do need larger premises. Plus we are growing all the time and that is good, but we will not fit in the end.
Good relations with stakeholders	…satisfied patients and that each patient is unique…that you both see and listen to each patient. They should feel like they are the only patient we have.

### Ethics

The study received ethical approval from the ethical research committee of the School of Health and Welfare, Jönköping University, HHJ2015/1878-51.

## Results

Each of the well-functioning PCUs showed robust performance in each of the 13 HWE indicators, but challenges and difficulties were also present.

### Positive, accessible and fair leaders

The staff at the PCUs shared a largely positive view of their managers, which they had not always experienced with previous managers.

She is the best boss I’ve ever had; she is outstanding.

They described their current managers as positive, open, listening, fair, interacting, creative, daring to make decisions, and visionary. Short decision paths were combined with present, prestige-less and enabling leadership, where innovation and creativity were encouraged, even though the innovative streak could be experienced as too dominating by a few. The organisations were described as flat and non-hierarchical, with the exception of physicians who were described as ‘on top’.

### Skilled communication

All PCUs had structured meetings for information exchange with leaders skilled in communication and in creating structures for information channels. Short, daily, morning meetings were important for solving problems and for maintaining a positive social climate. Despite the organised meetings, some staff found there was insufficient information, especially in larger or expanding units and in units focusing on intra-professional meetings instead of a combination of intra- and inter-professional meetings.

The communicative climate was permissive, but only to a certain extent; expression of negative feelings or opinions was discouraged in the working group, and instead these were expressed furtively and only between individuals.

We have managed to create an environment where we do not actually whine. I do not know when I heard whining lately, so if you pick up something for discussion, yes, you may complain among yourselves, but not openly.

Another challenge was to be able to criticise colleagues’ in a constructive way; some staff said that they seemed to like each other too much in the group, which hampered open communication. A few cases of insufficient communication were described which had led to feelings of insecurity and avoidance of solving conflicts thoroughly.

### Collaboration and teamwork

Staff worked with collaborative problem-solving to achieve the best possible care for patients and experienced supportive leaders. Collaboration and teamwork were positive aspects of work, useful for patients and fun for staff.

Everybody is helpful and you do this in everyone's best interests, not for your own sake, but for everyone.

Small units supported collaboration through increased possibilities for spontaneous meetings. An enabling collaborative factor was recruitment of staff with high ability in teamwork and collaboration, which decreased hierarchical inter-professional structures.

You work in teams and have different skills; we do not have a hierarchical thinking, but everyone's knowledge is important and that we utilise that knowledge in the best possible way.

Challenges mentioned included being the only person in a specific profession, difficulties in finding time for mutual meetings and not everyone participating in the collaborative improvement work.

### Positive social climate

The social climate was positive at all the PCUs, but the solidarity was not self-evident. Shared values and shared beliefs in the organisation’s virtuousness increased the positive feelings of trust and joy in the workplace. Working towards a positive social climate in the work place was a task for the individual, the group and the manager. The PCUs had various activities to enhance the positive social climate, such as collective celebrations, common lunches, united social affirmation together with praise from the manager to the group and regular structured contact. Strategic recruitment of staff with the same core values and positive attitudes strengthened the positive climate.

We have recruited those who have, what shall I say, so to speak a gleam in the eyes and a will to do something. So that's what distinguishes us from the previous organisations I've worked in.

To be positive was experienced as oppressive if combined with oppressing negative events. Stress, absence of leadership, and conflicts, as well as starting the day with negative feedback, were found to affect the social climate negatively.

### Employee involvement

The staff at each PCU presented a feeling of ‘us’ as a homogeneous group, sharing the same core values, striving for their best work performance to ensure good primary care for the patients. All units demonstrated a strong joint driving force to reach goals, creating employee involvement, team spirit and a willingness to promote the organisation.

I think it's great fun if we reach the goals, you're looking like wow now we got over 75% on this, now we’ve got a full pot. That way, it's great fun.

At the same time, some did not have a clear picture of the goals of the PCU; the experience of being governed by finance was negative for patients. A feeling of decreasing employee involvement was a challenge in growing organisations.

### Autonomy/empowerment

Staff shared a sense of expectation to take responsibility for their work in combination with the leaders providing trust in professional autonomy.

There is a shared responsibility in the group and you expect everyone to take their responsibility.

Nevertheless, frustration was created when the values, wishes or needs of individual staff members or patients conflicted with the financial goal or the daily structure of the work.

### Role clarity with clear expectations and goals

Clear role descriptions supported the work, but new financial incentives had led to changes in tasks and roles, which had created unclear expectations as well as possibilities for professional development. Lack of role descriptions and changes that decreased responsibility in a professional role were experienced as creating a feeling of ‘us against them’ in the group and conflicts regarding who was responsible for ‘attractive’ tasks. Vague and diversified roles involving a need to perform several tasks (a common occurrence at smaller PCUs) could create feelings of fragmentation and inadequacy.

If you have several tasks in your role, like I have had three for a while, it divides your attention so much, that it doesn’t work so well.

To have several roles in the workplace was a challenge for both the individual and the co-workers; feelings of safety and trust could be enhanced by clear expectations and humbleness concerning the knowledge of other professions.

### Recognition

Experiences of recognition included being respected for your work and for doing your best. Being able to help patients was experienced as essential and joyful. The staff felt recognised by their manager, by their colleagues and by the patients, and the feeling of recognition was further promoted by a good salary, good working hours, benefits (e.g. free breakfast) and having their own responsibilities. Accomplishment at work was important, and for competitive employees, it was rewarding to perform well at work; feelings of self-assurance were obtained by succeeding in one’s tasks.

I've always liked it and enjoyed challenges. You may feel that it's hard to do it, but once you've done it, you feel satisfied and feel “Oh! I did it”, and then you get huge energy from this.

Some PCUs had development conferences at nice locations on weekends, but work-related activities, when they were organised in leisure time, were experienced as extra work rather than a reward.

### Growth and development of the individual at work

The combination of personal and developmental drive gave the professionals opportunities for on-the-job professional development, e.g. change to more difficult or challenging tasks.

I have a great opportunity to develop in what I think is interesting.

Seeing others work hard for the organisation motivated the individuals to increase their efforts. The availability of external courses and training differed among staff and possibilities for formal competence development had decreased due to increased productivity demands and a decreased course budget. This led to *s*taff expressing feelings of guilt when they were away from the workplace on courses because this increased the work load for others and reduced productivity.

### Moderate work pace and workload

The work pace and workload varied, but were mainly experienced as tiring, which raised the question of whether the resources were directed towards the right things in the organisation. Personal responsibility for adaptation of work pace, especially in the case of vacancies, was a reason for tiredness, stress and worry, and this was an area where managerial support and appropriate staffing were presented as important. To ensure productivity, one PCU had all their competence development meetings scheduled at lunch time with food provided. This was positive for some but also experienced as a decrease in breaks. Increased phone access for patients and administrative work and decreased time for sharing patient information between professions created worry about patient safety, as well as stress, frustration and irritation.

There is not quite enough time to do it in a good way. Yes, that bothered me a lot. I may not have time to reflect fully or check things out as much as I would like. And I also cannot take those extra seconds needed to make the meeting with the patient a little more personal.

One PCU used strategic recruitment of staff, choosing persons capable of handling high demands at work.

### Administrative and/or personal support at work

The overall managerial support was positive and strong, but there was a need for additional support. For administrative staff, extra staff were hired when needed, but this was not as easily done for other professions. Other personal support was given in the group by communication and by ‘helping each other’.

I get full support from the manager, so we discuss how to do it together.

The PCUs were characterised by a listening and supportive attitude, even if professionals, said that the level of service to help them carry out their work had decreased. One PCU had used external consultants, which was experienced as unpleasant rather than supportive.

### Safe physical work

Not all PCUs could choose their own premises, which affected their ability to grow further and also affected the physical work environment in a negative way.

There is a lack of space and so, really we do need larger premises. Plus we are growing all the time and that is good, but we won’t fit in the end.

### Good relations with stakeholders

There was a strong collaborative culture among the PCUs regarding the importance of patient accessibility and thoughtful treatment of patients, together with strong core values of the organisation. There had been an increased focus on the patient as a customer and on the importance of treating patients well, leading to changes in working procedures.

…satisfied patients and that each patient is unique…that you both see and listen to each patient. They should feel like they are the only patient we have.

Despite positive consequences for patients, the view of patients as a means to create economic gain was experienced as negative by the staff.

## Discussion

The workers experienced all indicators of a healthy working environment (HWE) which means that a healthy work environment seemed to be in place at these well-functioning PCUs.

The positive experiences of HWE indicators at the PCUs were not self-evident but rather something the PCUs continuously worked hard to achieve. Negative experiences related to the indicators were present for all PCUs, showing that even well-functioning PCUs had challenges, which is in line with previous research on primary care [20]. It is important to acknowledge the complexity of organisations and that even well-functioning units can have dysfunctional aspects [21]. Many of the challenges experienced were common among the PCUs, such as lack of staff and many demanding patients needing care. The challenges varied, however, depending on whether the PCU was striving to grow or wished to remain with the current number of patients. Organisational growth, if executed at a high pace, was described as problematic and led to a decrease in HWE indicators. The results showed that HWE indicators are dependent on context and organisational culture, confirming that more or less all health environment factors need to be integrated in an organisational culture to promote healthy work places [22].

The discussion focuses on the similarities in the descriptions of the PCUs, and on their uniqueness in relation to the indicators. Three aspects are used to structure the discussion because they capture the experiences of the indicators and can be seen as resources that enhance an HWE: organisational virtuousness, employee commitment and joy at work.

Organisational virtuousness is what the organisation aspires to when performing at its very best, and the PCUs had different aspirations: productivity, societal well-being or shared religious beliefs. Previous research has shown significant relationships between organisational virtuousness and organisational performance [23]. Organisational virtuousness creates self-reinforcing positive spirals, together with a buffering function towards negative challenges to the organisation [23,24]. It is essential for organisations in primary care to work to enhance their cultural core values, especially if optimal performance includes task delegation, but also to acknowledge that changing a culture demands time and resources [25]. Several of the explored HWE indicators relate to factors associated with the development and support of organisational virtuousness such as fostering respect, integrity, gratitude, compassion, forgiveness, inspiration, and meaningful work [23].

Leadership is an important factor in HWE [12,26], as shown in this study. The positive experiences of the manager were important, because the opposite can create a climate of blame-shifting and distrust, undermining teamwork and enabling disrespectful treatment of patients [27]. The manager can play an important role in motivating, enhancing positive relations and buffering possible negative consequences of reforms at the staff level [18,26,28,29]. Positive practice may provide an important arena for leaders of organizations to enhance their organizations’ performance [30].

Skilled communication is an example of where not only the manager’s role but also the individual’s own role and behaviour are essential in creating an HWE [31]. Effective decision making affects HWE, and requires that the individual gets not only great responsibility but also the ability to influence the decisions that have an impact on the work [13]; improvement of job control is beneficial to workers’ health [14].

Employee commitment was shown through employees wanting to give their individual best possible performance to help the organisation work optimally and may be the main reason why the PCUs in this study functioned well. This is discussed in depth by Kjellström et al. [18] in terms of work motivation. It is a demanding but important aspect of management to boost the staff’s optimism when meeting difficulties, and strong organisational virtuousness and dedication among employees are valuable assets in this process [23,24,32,33].

To create a healthy work place and ensure a positive social climate, the effort to enhance good feeling is a worthwhile investment for the organisation, because this resource can lead to both increased and sustained well-being for the organisation and the individual [15,23,33]. If staff have a gloomy perception of their workplace, job demands are perceived as more burdensome, and negative information or situations may arise more often [14]. A positive social climate in the workplace can also enhance health by providing a positive context for patients at the PCU [34]. A non-permissive attitude towards being negative or expressing negative feelings can be problematic, because there is a risk for collective silence, which may lead to mistrust and reduced organisational performance [22]. Negative events have a greater impact than positive events, emphasizing the importance of a positive practice at the workplace [23].

Joy at work was common and based on experiences of personal mastery and feelings of meaningfulness but was also described as enjoyment of the positive and inspiring social culture in the workplace. Intrinsic motivation is connected to feelings of accomplishment and joy at doing well at work [35], and job-satisfaction is also related to a high degree of task delegation and good quality care [25]. Previous research has shown that intrinsic motivation decreased after the implementation of governance structure reforms in primary care [3], indicating that the well-functioning PCUs had other qualities that supported intrinsic motivation and joy at work. Frequent forums for communication, shared care models, team work and professional satisfaction have been found to influence joy at work among physicians in well-functioning primary care practices [20].

High workload and stress are related to negative effects both for the individual and the organisation. Health care professionals are a group that is highly vulnerable to work stress, and this may affect patients in a negative way. This study shows that joy at work can be present, despite high workloads. This finding is in line with previous studies on nurses [36] but is also in line with research presenting occupational stress as a motivator and a creator of feelings of accomplishment and personal satisfaction [37].

In our study, joy at work was experienced through accomplishment of work tasks and being part of a positive social community, but also through working with patients, which has been described in previous research [38]. Social support contributed to productivity and customer satisfaction, with a positive effect on decreasing workers’ sick leave and stress, thus enabling continuity of care [37,39]. In our study, several important coping skills are mentioned, such as seeking social support and engaging in problem-solving. Such skills may contribute to lessening the negative impact of a stressful work environment.

### Methodological discussion

The large multi-professional interview sample, with various professional backgrounds, strengthens the credibility of the results. A weakness of the study was the involvement of the manager in the sampling process which may have affected the results, but the risk is lessened by the large sample from each PCU. The concept of “well-functioning” is complex and the research group is aware that the description used is inadequate in capturing such a complex concept. In addition, organisations are in continuous movement, during the time passing between the acceptance and planning of the study and the data collection, organisational changes occurred for one of the included PCUs which was without a leader for a time, while three of the included PCUs have been the only units in the region with the same leaders since the reform in 2010. These changes point to the importance of discussing also sustainability when referring to an organization being well-functioning. Nevertheless, the concept is adequate as a way of describing this sample in this study, although we acknowledge the need to examine our results in a wider sample of PCUs. Additional studies exploring the HWE indicators at PCUs not defined as well-functioning would also be of interest for further research Transferability is enhanced by the variety of the PCUs included. Even though they were situated in the same region, the results may be transferable to similar primary care contexts. Most of the studies included in the review by Lindberg and Vingård [10] are from North America, and therefore the results from the Swedish context contribute to knowledge of the usefulness and transferability of the matrix.

## Conclusions and implications

The PCUs included in this study seemed to have healthy work environments in place based upon the experiences of its indicators. The PCUs thereby shared a similar pattern of work environment indicators, but at the same time they presented unique workplaces, with unique solutions and coping strategies to meet shared challenges. Even though the PCUs demonstrated healthy work environments, each of the indicators also presented challenges that needed to be dealt with. Staff at PCUs are thus encouraged not only to actively work to create and sustain HWE indicators but also to work actively to meet challenges at the workplace. The results suggest that useful approaches for a healthy work environment could be to address issues of organisational virtuousness, employee commitment and joy at work.
